# The potential effect of technology and distractions on undergraduate students’ concentration

**DOI:** 10.12669/pjms.334.12560

**Published:** 2017

**Authors:** Najya A. Attia, Lubna Baig, Yousef I. Marzouk, Anwar Khan

**Affiliations:** 1 Dr. Najya A. Attia, MD. King Saud bin Abdulaziz University for Health Sciences, Jeddah, Saudi Arabia; 2 Prof. Dr. Lubna Baig, Dean, APPNA Institute of Public Health, Jinnah Sindh Medical University, Karachi, Pakistan; 3 Dr. Yousef I. Marzouk, MD. King Saud bin Abdulaziz University for Health Sciences, Jeddah, Saudi Arabia; 4 Dr. Anwar Khan, MD. King Saud bin Abdulaziz University for Health Sciences, Jeddah, Saudi Arabia

**Keywords:** Cell phones, Classroom, External distractions, Internal distractions, Laptops, Medical students, Technology

## Abstract

**Background and Objectives:**

In the present era, it is difficult to keep the concentration of college students at its maximum potential during the class time, as there are many distractions that negatively impact students’ concentration and prevent optimal learning. Technologies such as laptops and cell phones have invaded the classroom, raising considerable concerns about their effects on college students’ attention in the classroom. Despite these concerns, no research has been done in Saudi Arabia on the effects of technology and other types of classroom distractions on students’ concentration. In the current study, we have attempted to identify students’ perceptions of major distractions in the classroom based on seventeen internally (self-produced) and twenty-four externally produced classroom situations.

**Methods:**

The students participating in this study rated the degree to which each distraction interferes with their concentration on the class materials and their ability to learn. Data were collected through surveys of 265 students (66 and 199 students from medical and basic classes, respectively), including 97 females and 168 males 17–23 years of age from the academic years 2010 to 2014. A validated self-administered questionnaire was handed to the students in the classroom. The students were asked to report and rate the classroom distraction produced by 24 external internal distracters (Table-II), on a 5-point scale.

**Results:**

The results revealed that ringing cell phones in the class were the most commonly reported electronic external distractor for 68% of students, and 21% of them reported being extremely distracted by this noise. Having an instructor who is difficult to understand was the most commonly reported external behavioral distractor for 75% of students, and 48% of them rated this as extremely distracting. Students talking in class were the most self-produced distractor for 72% of students; negatively impacting their concentration and ability to learn, and 42% of them rated it as an extreme distractor. Wearing clothing with unusual words, drinking and eating in the classroom were minimally distracting colleagues. Overall, distractions (internal and external) were more significant for fifth-year students than the other years at a p-value < 0.001.

**Conclusion:**

Students believed that laptop and cell phone use in the classroom can effect their concentration and ability to learn. The students also felt that inappropriate behavior is a major distraction for students as well, and thus necessitates monitoring and improvement.

## INTRODUCTION

It is difficult to keep the concentration of students at its maximum potential during the entire time of the class, as there are many distractions that can have a negative impact on students’ concentration and learning. Various forms of technology, such as laptops, cell phones, net books, tablets, and smart phones, have invaded the classroom.^1^ There has been considerable discussion in recent literature about the potential negative effects of various technologies on students’ concentration in the college classroom.^2^,^3^ Thishas led to some instructors and universities banning the use of electronic devices (cell phones, laptops) during class.^4^ Fried studied laptop use during class to determine its effects on student learning and found that it negatively affects students’ performance and learning.^3^ In addition, Granberg and Witte found that students’ use of laptops in the classroom does not improve their grades.^5^ Ironically the movement and lighting of text and pop-up messages in laptops have been found to reduce students’ performance and increase the number of errors.^6^ In a controlled study, students with open laptops remembered less lecture content than those with closed laptops.^7^ In contrast, Driver and Stephens have found that use of laptops in the classroom can enhance academic achievements and satisfaction of students.^8^,^9^

Cell phones are another attractive device that can affect students’ attention and concentration in the classroom, as students can be easily distracted by text messages and feel the urge to reply instantly. Shelton et al found significant negative effects of cell phone ringing on cognitive performance.^2^ A few researchers have found that the students’ use of cell phones in the classroom could distract both faculty and students.^1^,^10^ A study by the National Education Association demonstratedthat 85% of higher education instructors in the U.S. agreed that professors should ban cell phone use in the classrooms.^1^ Many educational institutions in the U.S. have enacted policies banning cell phone use in classrooms.^11^,^12^ It is important to note, however, that not all cell phone use in educational contexts is objectionable. Katz reported the potential positive effects of the technology for accessing Internet resources, tutoring, and connecting instructors, students, and parents coordinating school activities.^13^

Students’ and instructors’ behaviors and the classroom environment could be sources of distraction for students and negatively impact their learning. Meyers in the year 2003 classified student misbehavior as being either covert or overt.^14^ Covert behaviors are more passive and include arriving to class late, leaving class early, sleeping during class, or acting bored and disengaged. Overt behaviors are more open and include students eating or drinking noisily, talking during class, or using their cell phones. In 2005 a scientist sent a learning survey to 243 recent graduates asking them what inhibited their learning while in college; they responded that disruptive student behavior in the classroom inhibited their learning. He found that disruptive student behavior in the classroom not only inhibits student learning, but also impacts student retention. 15

The technical infrastructures of King Saud bin Abdulaziz University for Health Sciences (KSAU-HS) are adequate for laptop use, and the majority of students own laptops and cell phones, which are not prohibited from being used during class time. In general, laptop and cell phone use in the classroom and students’ disruptive behavior raise considerable concerns about their effects on students’ performance. No research in Saudi Arabia has been done to-date on the effects of these technologies (laptops and cell phones) and other types of classroom distraction on students’ concentration and performance. The current study aimed to study the perception of students regarding effect of technology and disruptive behavior on concentration and learning process. The study also explored the student’s perceptions of external and internal (self-produced) distractions in the classroom.

## METHODS

### Study design: Quantitative exploratory study Sample

A sample of 200 students was identified using 5% margin of error, expected frequency of cell phone use at 80% and confidence level of 95% by using Raosoft^®^ software by the website www.raosoft.com/samplesize.html. Convenience sampling was used and all students present in the class at the time were given the questionnaire. The research was conducted in classrooms where laptops and cell phones were not utilized in an organized fashion. All students in each class had laptops and cell phones with wireless networking capabilities, and classrooms were equipped with Wi-Fi.

Two hundred sixty-five students from medical and basic science classes who were 17–23 years of age, from the academic years 2010–2014, participated in the research. There were 22 students from 5^th^, 26 from 4^th^ and 18 from 3^rd^ year (total 66) of medical college (Table-III). There were 199 students from the basic science college. All participants signed consent forms and were assured by the investigators that all data would be kept confidential and that the survey results would not influence their grades.

**Table-I:** Mean of distraction use among different Academic Groups.

*Mean*	*Year 5 5(n=22)*	*Year 4 (n=26)*	*Year 3 (n=18)*	*Year 2 (n=85)*	*Year 1 (n=114)*	*p-value*
External distract	3.0±0.7	2.6±0.4	2.7±0.5	2.5±0.7	2.5±0.6	0.001
Internal	3.4±0.9	2.9±0.7	3.2±0.8	2.5±0.9	2.5±0.9	<0.001

**Table-II:** External Distracters.

		*Mean±St. Dev.*
1	Ringing cell phones	2.8±1.44
2	Students texting	2.4±1.51
3	Students using video games	2.8±1.64
4	Students using smart phones	2.2±1.54
5	Students using MP3 players	2.3±1.61
6	Students using laptops for email, surfing net	2.1±1.42
7	Students talking with others in class	3.6±1.60
8	Poor personal hygiene of other students (odors, looking dirty, etc.)	2.9±1.69
9	Students asking irrelevant questions or making irrelevant comments	3.2±1.66
10	Students making repetitive movements (tapping fingers, pen clicking, etc.)	2.9±1.53
11	Student illness symptoms (coughing, sneezing, sniffling, etc.)	2.5±1.41
12	Students arriving late	2.2±1.36
13	Students leaving early	2.0±1.39
14	Students leaving/returning to class	2.5±1.49
15	Students eating in class	2.2±1.46
16	Students drinking in class	1.7±1.31
17	Students sleeping	2.0±1.53
18	Students doing work for other courses	2.0±1.49
19	Instructor that is difficult to understand	3.5±1.62
10	Instructor exhibiting repetitive or unusual movements	2.9±1.51
21	Instructor using repetitive words or phrases	2.7±1.52
22	Furnishings (e.g., chairs, tables that are broken, dirty, etc.)	2.7±1.60
23	Classroom odors equipment problems (e.g., malfunctioning computers)	3.2±1.67
24	Temperature (too hot/cold)	3.5±1.63

**Table-III:** Self-Produced Distracters.

		*Mean±St. Dev.*
1	Your phone ringing	2.9±1.59
2	Using your smart phone	2.9±1.63
3	Playing video games	3.1±1.75
4	Using your MP3 player	2.7±1.78
5	Texting during class	3.0±1.67
6	Using a laptop for checking your email, surfing, etc.	2.7±1.65
7	Doing work for other courses	2.9±1.68
8	Talking with others in class	3.3±1.63
9	Arriving late to class	2.9±1.64
10	Leaving early	2.6±1.65
11	Leaving/returning to class	2.7±1.62
12	Eating in class	2.2±1.51
13	Drinking in class	1.8±1.37
14	Sleeping in class	2.8±1.80
15	Poor personal hygiene (odors, looking dirty, etc.)	2.7±1.70
16	Your illness symptoms (coughing, sneezing, sniffling, etc.)	2.8±1.57
17	Wearing clothing with unusual words, colors, styles, etc.	2.0±1.48

A validated self-administered questionnaire was handed to the students in the classroom. The students were asked to report and rate the classroom distraction produced by 24 external distracters (Table-I) and 17 internal distracters (Table-II), on a 5-point scale. The options for the external distracters included the use of laptops or cell phones by other students in class, other students’ behavior, instructors’ behavior, the classroom environment, or other. The options for the internal distracters included the use of laptops or cell phones by students in class, arriving late to class or leaving early, talking with others in class, eating or drinking in the class, and/or any other. The degree of distraction and the percentage of students who were distracted by instructors and class material determined the impact of technology use and distraction in the classroom on the students’ learning.

## RESULTS

Only the students who completed both parts of the questionnaire were included in the analysis. Two hundred sixty-five students out of 293 completed the surveys, which gave an overall response rate of 90.4%.

This study as described in the method section measures the student’s perception of distraction by technology and, external and internal distracters. Participants rated the degree of different classroom distracters that could interfere with their concentration in the class and learning. The survey asked 55different aspects of the classroomdistractions, which included external and internal (self-produced) classroom distraction.

**Fig. 1:**
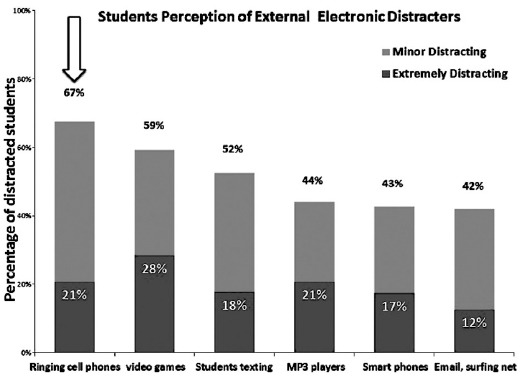
External electronic classroom distracters.

**Fig. 2:**
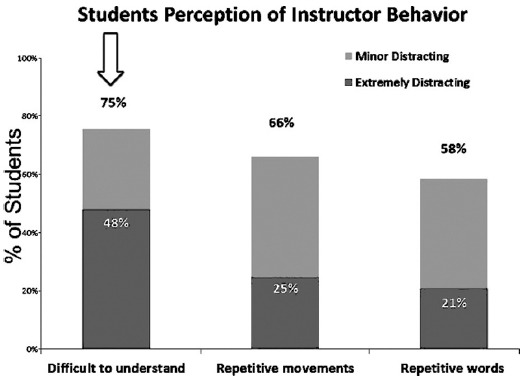
Instructor behavior distracters.

**Fig. 3:**
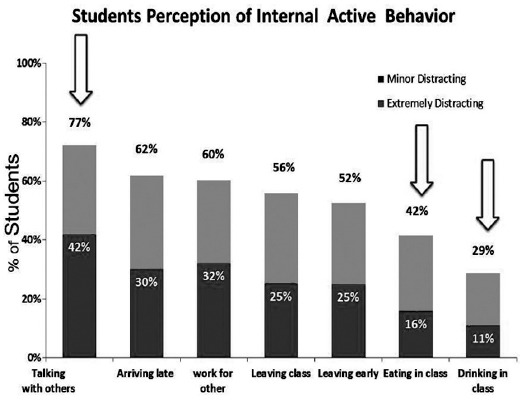
Internal active behavior distracters.

**Fig. 4:**
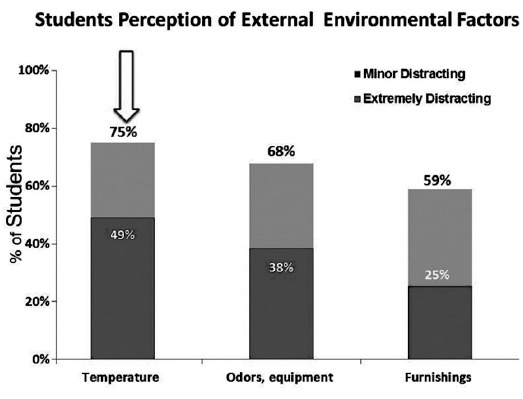
External enviromental classroom distracters.

The results indicated that, 67% of students reported cell phone ringing in the class as the most electronic external distractions that interfered with their concentration and learning ability of material presented in class and was extremely distracting to 21% of the them (Fig. 1). Forexternaldistracters 75%of students rated an instructor who is difficult to understand as mostdistracting and out of these 48% said that they were extremely distracted (Fig. 2). Seventy seven percent of the students said that talking of other students distracted them, 42% of them were extremely distracted (Fig. 3). For classroom Environmental Factors, abnormal classroom temperature (too hot/cold) was a distraction for 75% of students followed by malfunction of equipment 68% and Furnishings 59%(Fig. 4).

A comparison of the senior students (fifth, fourth and third year) with junior (second and first year) indicated an interaction on the distracters. The senior students were significantly more distracted than the juniors (Table-I).

## DISCUSSION

The, main findings of the study were that 67% of students were distracted by use of cell phones and 21% of them were extremely disturbed and it affected their learning. The External stimuli that effected students learning included 24 items (Table-II). Their internal stimuli that effected student learning included 17 items (Table-III). Our study is in concurrence with international studies that have shown that the use of laptop in the class was distracting to fellow students particularly with respect to flushing of instant message, and searching websites.^3^,^16^

The students reported that cell phone ringing in the class was a major distractor towards their learning. This implies that the laptop and cell phone use in the classroom can impact negatively on the students’ learning process. The educational institutions are for imparting knowledge and enhancing students learning experiences, henceforth if there are distracters, which can be removed conveniently, then appropriate policies should be in place for their judicial use. Keeping the phone on silent can be strictly followed to ensure minimal distraction and the faculty can start by role modeling that behavior.

Our study also identified that the instructor who is difficult to understand is also a major external distractor for majority of the students and hampers their attention towards learning similar to study by Fried who found that an instructor that is difficult to understand is one of the top four external stimuli.^3^ Thisfinding highlights the importance of instructors knowledge of the principles of teaching and methods for enhancing students concentration in the class.

The students also identified that talking to each other in the class disturbs them;Fried (2008) also found that students talking with others in class is the second common external stimuli.^3^ Poor personal hygiene, students talking with other students in class and illness symptoms were perceived to be distracting thestudents. In contrast, wearing clothing with unusual words, drinking and eating in the classroom were minimally distracting the students. This could be because the university has a strict dress code and faculty generally abides by them.

Instructors through setting up class rules and asking student to act professionally can manage majority of external stimuli inclusive of students’ behavior. The other external stimuli like unwanted noises, temperature, teaching resources etc. were also identified as distracters effecting student learning. This can also be adequately managed by the office staff and instructorsby checking equipment, temperature and source of noise before starting class and reporting any other possible distractor to the management.

The current study found a significant higher off-task behavior and rate of distraction in senior students than junior students which indicates that the freshman students are more attentive than senior students. It could be because most of information they receive are new or they adapt to the distractions from school.

### Limitations

One of limitation of the study was that it was done in a single institution and may not adequately reflect on the behavior in other institutions. However we believe that given that these students are from the general population of students from KSA henceforth the distracters may be very similar. This study is from a health science university however we believe that the results from other universities may not be varying significantly. Therefore, further research is needed to confirm our findings in other universities and disciplines.

## CONCLUSION

The use of laptops and cell phones in class had some negative effects on student learning. As we did not investigate the nature of use henceforth we may say with caution based on our results that use laptops and cell-phones in class do cause distraction This distraction could affect the ability to learn in the class. Therefore the university should enact policies for judicial use of digital devices during class time.

## FUTURE RESEARCH

Our future research will describe the nature of laptop and mobile phone use in classroom. We also recommend that other institutions in KSA from other disciplines should do similar studies to have broad term policies in-effect, which would hopefully enhance students learning in all disciplines.
